# Thoracoabdominal Aortic Aneurysm Repair Using Fenestrated and Branched Endovascular Grafts for High-Risk Patients: Evolving yet Safe

**DOI:** 10.1177/15266028241229005

**Published:** 2024-02-10

**Authors:** Daniyal N. Mahmood, Rodolfo Rocha, Maral Ouzounian, Kong Teng Tan, Samantha M. Forbes, Jennifer C-Y. Chung, Thomas F. Lindsay

**Affiliations:** 1 Division of Vascular Surgery, Department of Surgery, Peter Munk Cardiac Centre, Toronto General Hospital, University of Toronto, Toronto, ON, Canada; 2 Division of Cardiovascular Surgery, Peter Munk Cardiac Centre, Department of Surgery, University of Toronto, Toronto, ON, Canada; 3 Division of Interventional Radiology, Toronto General Hospital, Toronto, ON, Canada

**Keywords:** FEVAR, TEVAR, BEVAR, thoracoabdominal aortic aneurysm repair, endovascular aneurysm repair

## Abstract

**Purpose:**

The purpose was to investigate outcomes of high-risk patients undergoing thoracoabdominal aortic aneurysm (TAAA) repair using fenestrated or branched endovascular aneurysm repair (F/BEVAR) devices at a single center in Canada.

**Materials and Methods:**

A retrospective review of all patients undergoing endovascular TAAA repair with F/BEVAR from June 2007 to July 2020. Imaging and clinical endpoints of interest including death, reintervention, and target vessel patency were reported.

**Results:**

Ninety-five consecutive patients underwent endovascular TAAA repair using F/BEVAR stent grafts (63 males, median age 74 [interquartile range 70, 78] years). Repairs included 81 elective and 14 urgent/emergent cases (6 ruptures and 8 symptomatic). Graft deployment was 100% successful. Intraoperative target vessel revascularization was successful in 336/355 (94.6%) vessels with the celiac having the lowest success rate 72/82 (87.8%). In-hospital mortality was 9.5% (7.4% elective and 21.4% urgent/emergent, p=0.125) and permanent paraplegia was 4.2% (3.7% elective and 7.1% urgent/emergent, p=0.458). In-hospital complications included stroke in 5.3%, acute myocardial infarction in 8.4%, and bowel ischemia in 5.3%. No patients required permanent dialysis or tracheostomy during their hospital stay. However, 22 (23.2%) patients required additional unplanned procedures for various indications (branch occlusion, endoleaks, realignment) during their hospital stay. Patients were followed up for a mean of 3.6 ± 3.0 years. Clinical follow-up was 100%, with 80/86 (93%) having surveillance imaging. On follow-up imaging, 43 (50%) patients had at least 1 endoleak identified and 337/341 (98.8%) of the target vessels were found to be patent. At 5 years, cumulative probability of reintervention was 46.3% (95% confidence interval [CI], 36.1-56.4). Survival at 5 and 8 years was 50.1% (95% CI, 38.4-65.4) and 34.4% (95% CI, 22.5-52.8), respectively. Progression of aneurysmal disease leading to rupture on follow-up was confirmed in 1 patient at 10 years.

**Conclusion:**

Endovascular TAAA repair provides a safe treatment option with a high technical success rate and low pulmonary and renal complications. Long-term survival is similar to previous literature; however, high rates of secondary reintervention reaffirm the need for ongoing patient follow-up and further technical improvements.

**Clinical Impact:**

This study demonstrates that endovascular repair of TAAAs can be performed in a high-risk elderly population with acceptable rates of mortality, TALE and SCI, using evolving technology. The incidences of post-operative respiratory failure and renal dysfunction were lower in patients who underwent endovascular repair compared with open repair. Future technical and procedural refinements in addition to increasing surgical experience are expected to lead to further improvements in short- and long-term outcomes exceeding those of open repair.

## Introduction

Thoracoabdominal aortic aneurysm (TAAA) repair using fenestrated/branched endovascular aneurysm repair (F/BEVAR) devices was initially described by Chuter and colleagues in 2001.^1^ It provided an alternative treatment modality for patients who were deemed to be high surgical risk for open repair, even at high-volume centers.^2–4^ Successful trials of infrarenal endovascular aneurysm repair (EVAR) for AAAs in the 1990s^5^ paved the way for minimally invasive techniques to treat more complex aneurysms. Multiple meta-analyses^6,7^ have identified an early survival benefit with infrarenal EVAR, with a higher rate of reintervention but unchanged long-term survival compared with open repair.^8^ For TAAA, literature to date has found similar rates of midterm mortality, permanent paraplegia, and other comorbidities between open and endovascular repair.^9–12^

A systematic review has documented excellent outcomes following F/BEVAR for TAAA repair^10^; however, there is a paucity of real-world data evaluating its midterm outcomes while including evolving techniques that minimize spinal cord ischemia (SCI). Furthermore, new reporting standards allow for more objectivity when comparing outcomes of endovascular repair between centers of different referral volumes.^13^ This study aims to report a thorough and evolving endovascular TAAA experience from a single center.

## Materials and Methods

### Study Population

All 95 consecutive patients from a tertiary center treated for TAAA using F/BEVAR from the initiation of the program in November 2007 to June 2020 were identified from an institutional endovascular database and included. Patients with aortic aneurysms involving the renal-mesenteric arteries were seen in a multidisciplinary aortic clinic and physiological fitness was assessed by criteria adopted from Society for Vascular Surgery practice guidelines.^14^ Our selection criterion for elective patients includes, but is not limited to, maximum aneurysm size >6 cm, no active malignancy, normal pulmonary function tests with forced expiratory volume >1 L, cardiologist clearance with left ventricle ejection fraction >40%, an agreement to attempt cessation/reduce smoking, and no active incidental pathologies found during investigations. All elective, symptomatic and ruptured aneurysms were included. During this time period, 62 open TAAA repairs were performed. All patients in this series were deemed to be high surgical risk for open repair and consented for endovascular repair. All computerized tomography (CT) relevant angiograms were reviewed for inclusion criteria, using Coral (hospital-based picture archiving and communication system) and TeraRecon (ConcertAI, Durham, North Carolina). The Crawford-Safi classification and the extent of aortic coverage were separately measured and recorded.^15^ Indications for surgery were based on aneurysm size and rate of growth and the study was approved by the Institutional Research Ethics Board.

### Procedure Details

This report includes the learning curve and experience of 8 surgeons over the 13 years. All procedures were undertaken in hybrid operating rooms, which began with Toshiba Aquilion Imaging (Tokyo, Japan), then a Siemens Artis Zeego (Munich, Germany), and most recently a Philips Azurion (Amsterdam, the Netherlands), that latter 2 systems had fusion imaging. Preoperative assessments were recorded including open or percutaneous procedures to facilitate endovascular TAAA repair. Intraoperative arterial access was via arterial cut-down initially, with occasional conduit use, and evolved to percutaneous access. Endografts were either customized fenestrated or branched grafts or an off-the-shelf T-branch (Cook Medical, Australia) graft. Directional branches were employed for longitudinally oriented target vessels and when the distance between the stent graft and aortic wall >5 mm, while fenestrations were employed for transversely originating target vessels where the main graft was planned to reach the aortic wall (<5 mm). Bridging stents began with Advanta V12 (Atrium Medical Corp, Hudson, New Hampshire), Gore Viabahn (W.L. Gore & Associates, Flagstaff, Arizona), and Fluency (BD, Franklin Lakes, New Jersey). This then transitioned to also include VBX (W.L. Gore & Associates) and Lifestream (BD). EPIC uncovered stents (Boston Scientific, Marlborough, Massachusetts) were occasionally used to prevent branch kinking. Twenty-five target vessels did not require a branch/fenestration due to prior occlusion (n=9), nephrectomy (n=4), renal atrophy (n=4), bypass (n=3), or use of scallop (n=5). If a planned target vessel was not successfully cannulated intraoperatively, it was either reattempted as a planned reintervention or rarely occluded with an Amplatzer Vascular Plug (St. Jude Medical, St. Paul, Minnesota) if collateral supply was adequate. Proximal thoracic EVAR (TEVAR) and distal bifurcated grafts were used in patients to obtain adequate seal zones depending on aneurysm extent to achieve a 20 mm sealing zone in healthy aortic tissue, and the former was also utilized if at least 2 stent overlap was required with a staged TEVAR. The current protocol for minimizing SCI has evolved to include staged TEVAR which began in 2012, minimally invasive staged segmental artery coil embolization (MISACE) which began in 2017 and has been previously described by our group,^16^ permissive hypertension, intraoperative neuromonitoring of motor evoked potentials (MEPs) and somatosensory evoked potentials (SSEPs), early sheath removal with distal limb perfusion, baseline hemoglobin targets, hyperbaric oxygen therapy, and cerebrospinal fluid (CSF) drainage.^17,18^ Those who underwent both MISACE and TEVAR staging had MISACE first and TEVAR subsequently during a separate admission at least 2 weeks later.

### End Points

Primary endpoint was in-hospital and long-term mortality. Secondary outcomes include thoracoabdominal aortic aneurysm life-altering events (TALE), a composite of mortality, stroke, paraplegia, and permanent dialysis.^4^ Spinal cord ischemia is graded according to the 4-level system (grade 0-3), and acute kidney injury is defined as doubling of baseline serum creatinine.^13^ Primary technical success was defined as successful delivery and deployment of the aortic stent graft and its side branches on an intent-to-treat basis, target vessel patency on CT or duplex ultrasound (DU) imaging prior to discharge, and absence of type I and III endoleaks beyond 30 days. Assisted primary technical success included any unplanned in-hospital procedures required to meet technical success. Early postoperative period was to hospital discharge; events afterwards are included within follow-up.

### Follow-up

Clinical and imaging follow-up with either CT or DU took place 1, 3, and 6 months after discharge, and annually thereafter at the index hospital or a regional center. Follow-up imaging studies were reviewed to evaluate target vessel patency and endoleaks. Graft branches intentionally occluded to treat endoleaks were not deducted from branch patency rates. Follow-up was obtained through a combination of in-person clinic appointments or researcher phone calls using an approved standardized questionnaire.

### Statistical Analysis

All analyses were performed using the R project of Statistical Computing version 3.6.1. Continuous variables are described as mean ± standard deviation (SD) or median (interquartile range [IQR]), while categorical variables are represented as percentages. To evaluate differences between elective and urgent/emergent cohorts, student’s *t* test or Mann-Whitney tests were used for continuous variables, while the Fischer exact test was used for categorical variables. Time-to-event analyses for freedom from TALE and survival were performed using Cox proportional hazards models to obtain hazard ratios 8 years after surgery. Long-term rates of secondary procedures on the thoracoabdominal aorta or its branches were obtained accounting for death as a competing risk. Kaplan-Meier survival functions were estimated for survival and freedom from TALE. Cumulative incidence functions were generated for secondary interventions. A p<0.05 was deemed to be significant.

## Results

### Patient and Aneurysm Characteristics

Ninety-five consecutive patients (63% male; median age 74 [IQR 70, 78] years) underwent F/BEVAR repair for TAAA; 81 patients (85.3%) were treated electively, and 14 (14.7%) patients required an urgent/emergent procedure. Contained rupture was present in 6 (6.3%) patients and 8 (8.4%) were symptomatic (Table 1). Crawford extents were extent I: 3 (3.2%), extent II: 23 (24.2%), extent III: 27 (28.4%), extent IV: 37 (38.9%), and extent V: 5 (5.3%). Twelve patients (12.6%) had dissections and 2 (2.1%) presented with intramural hematomas.

**Table 1.** Preoperative Patient and Aneurysm Characteristics.

Variable	Patients, N (%)			p value
	Elective, n=81	Urgent/Emergent^a^, n=14	Total, n=95	
Age (years), median (IQR)	74 (70, 79)	74 (66, 77)	74 (70, 78)	0.417
Cardiovascular risk factors				
Cerebrovascular disease (stroke/TIA)	15 (18.5)	1 (7.1)	16 (16.8)	0.452
Dialysis	1 (1.2)	1 (7.1)	2 (2.1)	0.274
COPD	36 (44.4)	8 (57.1)	44 (46.3)	0.401
Coronary artery disease	32 (39.5)	5 (35.7)	37 (38.9)	0.99
Previous MI	16 (19.8)	2 (14.3)	18 (18.9)	1.0
Previous PCI	13 (16.0)	2 (14.3)	15 (15.8)	1.0
Previous CABG	10 (12.3)	2 (14.3)	12 (12.6)	1.0
Diabetes mellitus	14 (17.3)	1 (7.1)	15 (15.8)	0.457
Dyslipidemia	48 (59.2)	8 (57.1)	56 (58.9)	1.0
Hypertension	71 (87.6)	12 (85.7)	83 (89.2)	1.0
Peripheral vascular disease	25 (30.9)	6 (42.8)	31 (32.6)	0.374
Smoking (current or past)	69 (85.1)	10 (71.4)	79 (83.1)	0.245
Preoperative evaluation				
BMI (kg/m^2^)	28.6 ± 5.2	26.4 ± 5.1	28.3 ± 5.2	0.202
Baseline eGFR (mL/min/1.73 m^2^), mean ± SD	63.9 ± 21.7	69.1 ± 31.9	64.8 ± 23.6	0.525
CKD stage III-V	31 (38.3)	5 (35.7)	36 (37.9)	1.0
CKD stage IIIa	15 (18.5)	1 (7.1)	16 (16.8)	
CKD stage IIIb	11 (13.6)	2 (14.3)	13 (13.7)	
CKD stage IV	5 (5.3)	1 (7.1)	6 (6.3)	
CKD stage V	—	1 (7.1)	1 (1.0)	
Family history of aneurysms	14 (17.3)	2 (14.3)	16 (16.8)	1.0
Previous interventions				
Previous aortic surgery (n≥1)^b^	35 (43.2)	6 (42.8)	41 (43.2)	1.0
Root and ascending aorta	6 (7.4)	2 (14.3)	8 (8.4)	
Aortic arch	3 (3.7)	1 (7.1)	4 (4.2)	
Descending aorta	6 (7.4)	1 (7.1)	7 (7.4)	
TAAA	7 (8.6)	1 (7.1)	8 (8.4)	
Abdominal aorta	20 (24.7)	2 (14.3)	22 (23.2)	
Adjunctive open procedures pre-TAAA	8 (9.9)	0	8 (8.4)	0.599
H&N vessel debranching	2 (2.5)	—	2 (2.1)	
Elephant trunk arch repair	3 (3.7)	—	3 (3.2)	
Bilateral renal artery bypass	1 (1.2)	—	1 (1.0)	
Iliofemoral conduit placement	2 (2.5)	—	2 (2.1)	
Adjunctive percutaneous procedures pre-TAAA				
TEVAR staging	25 (30.9)	3 (21.4)^c^	28 (29.5)	0.752
MISACE	12 (14.8)	2 (14.3)	14 (14.7)	1.0
Aneurysm characteristics				
Maximum aneurysm size (mm), median (IQR)	68 (64, 72)	69 (62, 79)	68 (63, 72)	0.919
Crawford classification: aneurysm extent				
I	3 (3.7)	—	3 (3.2)	1.0
II	17 (21.0)	6 (42.8)	23 (24.2)	0.096
III	23 (28.4)	4 (28.6)	27 (28.4)	1.0
IV	33 (40.7)	4 (28.6)	37 (38.9)	0.555
V	5 (6.2)	—	5 (5.3)	1.0
Dissection	9 (11.1)	3 (21.4)	12 (12.6)	0.377
Acute	—	2 (14.3)	2 (2.1)	
Chronic	9 (11.1)	1 (7.1)	10 (10.5)	
Type A	2 (2.5)	—	2 (2.1)	
Type B	7 (8.6)	3 (21.4)	10 (10.5)	
Intramural hematoma	—	2 (14.3)	2 (2.1)	0.020
Contained rupture on presentation	—	6 (42.8)	6 (6.3)	<0.001
Symptomatic aneurysm	—	8 (57.2)	8 (8.4)	<0.001

Continuous data are presented as mean ± SD or median (IQR); categorical data are given as the counts (percentage).

Abbreviations: BMI, body mass index; CABG, coronary artery bypass grafting; CKD, chronic kidney disease, COPD, chronic obstructive pulmonary disease; eGFR, estimated glomerular filtration rate; H&N, head and neck; IQR, interquartile range; MI, myocardial infarction; MISACE, minimally invasive staged segmental artery coil embolization; PCI, percutaneous coronary intervention; SD, standard deviation; TAAA, thoracoabdominal aortic aneurysm; TEVAR, thoracic endovascular aortic repair; TIA, transient ischemic attack.

a Urgent mean time to operating room (7.8 ± 5.4 days), emergent (<24 hours).

b Some patients received more than 1 previous aortic surgery.

c Patients required urgent second stage repair after elective TEVAR staging.

p<0.05 is considered significant.

### Endovascular Repair

Twenty-eight patients (29.5%) received TEVAR staging, with the majority (n=22) in extent II/III aneurysms, placed a mean of 73 ± 94 days prior to primary TAAA repair, and landing a mean of 3.2 ± 1.4 cm above the celiac axis. Minimally invasive staged segmental artery coil embolization was performed beginning in 2017 in 14 (14.7%) patients at a mean of 51 ± 30 days prior to repair, embolizing on average 2.9 ± 1.4 segmental arteries per patient. Seven patients received both staged TEVAR and MISACE (Table 1).

Custom grafts were used in 79/81 elective patients, while T-branch grafts were used in 2 elective patients and 9/14 urgent/emergent patients (Table 2). Of the custom grafts, 45 (53.6%) were BEVARS, 27 (32.1%) FEVARS, and 12 (14.3%) F/BEVARs. A total of 355 visceral arteries were targeted with 231 (65.1%) directional branches and 124 fenestrations. Intraoperative proximal extensions were placed in 57.9% patients to ensure appropriate overlap. The total Crawford extent of coverage as defined per guidelines was extent I: 2.1%, extent II: 47.4%, extent III: 15.8%, extent IV: 31.6%, and extent V: 3.1%. Extent of graft coverage changed the classification in 31 (32.6%) cases. Graft delivery and deployment was 100% successful with no conversions. Target vessel revascularization was successful in 336/355 (94.6%) visceral arteries using a mean of 1.2 ± 0.4 (range: 1-3) covered bridging and 0.2 ± 0.4 (range: 0-2) self-expanding stents for kinks. No significant differences in target vessel revascularization were found between branches and fenestrations (94% vs 96%, p=0.43). Primary and assisted primary technical success was achieved in 72 (75.8%) and 82 (86.3%) patients, respectively. Assisted technical success was due to type I and III endoleak repair (n=8) and Celiac Artery (CA) revascularization (n=2). Unsuccessful visceral artery revascularization was responsible for the remaining technical failures in patients (n=13).

**Table 2.** Procedural Details for Endovascular TAAA Repair.

Variable	Patients, N (%)			p value
	Elective, n=81	Urgent/Emergent, n=14	Total, n=95	
Graft type				<0.001
Customized graft	79 (97.5)	5 (35.7)	84 (88.4)	
T-branch graft	2 (2.5)	9 (64.3)	11 (11.6)	
Distal extent	61 (75.3)	13 (92.8)	74 (77.9)	0.182
Bifurcated graft	46 (56.8)	7 (50.0)	53 (55.8)	
Straight graft	11 (13.6)	3 (21.4)	14 (14.7)	
Iliac branched graft	4 (4.9)	3 (21.4)	7 (7.4)	
Crawford classification: extent of repair				
I	2 (2.5)	—	2 (2.1)	1.0
II	35 (43.2)	10 (71.4)	45 (47.4)	0.080
III	13 (16.0)	2 (14.3)	15 (15.8)	1.0
IV	28 (34.6)	2 (14.3)	30 (31.6)	0.213
V	3 (3.7)	—	3 (3.1)	1.0
CSF drainage	67 (82.7)	14 (100)	81 (85.3)	0.120
Prophylactic	65 (80.2)	12 (85.7)	77 (81.1)	
Postoperative prophylactic	—	1 (7.1)	1 (1.0)	
Postoperative rescue	2 (2.5)	1 (7.1)	3 (3.2)	
Duration of drain in situ (days), median (IQR)	3 (2, 4)	3 (2, 4)	3 (2, 4)	0.922
SSEP/MEP neuromonitoring	36 (44.4)	—	36 (37.9)	<0.001
Amount of contrast used (mL), median (IQR)	209 (170, 283)	295 (172, 343)	220 (170, 295)	0.138
Fluoroscopy time (min), median (IQR)	121 (92, 148)	150 (110, 185)	125 (94, 155)	0.078
Successful visceral artery revascularization	288/301 (95.7)	48/54 (88.9)	336/355 (94.6)	0.052
Celiac artery	60/69 (87.0)	12/13 (92.3)	72/82 (87.8)	1.0
Superior mesenteric artery	78/79 (98.7)	13/14 (92.8)	91/93 (97.8)	0.280
Right renal artery	76/77 (98.7)	12/14 (85.7)	88/91 (96.7)	0.061
Left renal artery	74/76 (97.4)	11/13 (84.6)	85/89 (95.5)	0.100
Length of procedure, mean (min) ± SD	550.1 ± 108.5	654 ± 130.9	565.8 ± 117.4	0.013

Continuous data are presented as mean ± SD or median (IQR); categorical data are given as the counts (percentage).

Abbreviations: CSF, cerebrospinal fluid; IQR, interquartile range; MEP, motor evoked potential; SD, standard deviation; SSEP, somatosensory evoked potential; TAAA, thoracoabdominal aortic aneurysm.

p<0.05 is considered significant.

### Postoperative In-Hospital Course

In-hospital mortality was 9.5%: 7.4% in elective patients and 21.4% in urgent/emergent presenting patients (Table 3). Causes of overall in-hospital mortality included myocardial infarction (n=1), toxic megacolon (n=1), respiratory infection (n=1), stroke (n=2), multisystem organ failure with sepsis (n=1), intraoperative iliac rupture (n=1), and 2 patients ruptured postoperatively (1 from type Ia endoleak and 1 due to an unstented Superior Mesenteric Artery (SMA) [occluded between surgical planning and the procedure, rupturing the aneurysm prior to planned embolization]).

**Table 3.** Perioperative Events and Requirements.

Variable	Patients, N (%)			p value
	Elective, n=81	Urgent/Emergent, n=14	Total, n=95	
Hospital length of stay (days), median (IQR)	9 (6, 16)	19 (9, 24)	10 (6, 18)	0.052
ICU length of stay (days), median (IQR)	4 (3, 7)	7 (5, 12)	5 (3, 7)	0.011
In-hospital mortality	6 (7.4)	3 (21.4)	9 (9.5)	0.125
Extent of repair				
Extent I	0/2	—	0/2 (0)	
Extent II	2/35 (5.7)	3/10 (30)	5/45 (11.1)	
Extent IIII	2/13 (15.4)	0/2 (0)	2/15 (13.3)	
Extent IV	2/28 (7.1)	0/2 (0)	2/30 (6.7)	
Extent V	0/3 (0)	—	0/3 (0)	
Spinal cord ischemia (SCI)				0.458
Early SCI (<24 hours)	14 (17.3)	4 (28.6)	18 (18.9)	
Delayed SCI (>24 hours)^a^	7 (8.6)	2 (14.3)	9 (9.5)	
Temporary SCI (grade 1)	7 (8.6)	2 (14.3)	9 (9.5)	
Permanent paraparesis (grade 2)	9 (11.1)	1 (7.1)	10 (10.5)	
Permanent paraplegia (grade 3a-3c)	2 (2.5)	2 (14.3)	4 (4.2)	
Extent of repair (any SCI)	3 (3.7)	1 (7.1)	4 (4.2)	
Extent I	0/2	—	0/2 (0)	
Extent II	8/35 (22.8)	4/10 (40)	12/45 (26.7)	
Extent IIII	5/13 (38.5)	0/2 (0)	5/15 (33.3)	
Extent IV	1/28 (3.6)	0/2 (0)	1/30 (3.3)	
Extent V	0/3 (0)	—	0/3 (0)	
Stroke	2 (2.5)	3 (21.4)	5 (5.3)	0.022
Permanent dialysis	0	0	0	—
TALE	11 (13.6)	4 (28.6)	15 (15.8)	0.226
Aneurysm rupture	1 (1.2)	1 (7.1)	2 (2.1)	0.274
Acute kidney injury (>2-fold increase in Cr)	5 (6.2)	1 (7.1)	6 (6.3)	1.0
Transient dialysis	3 (3.7)	—	3 (3.2)	1.0
TIA	1 (1.2)	—	1 (1.0)	1.0
Acute MI	5 (6.2)	3 (21.4)	8 (8.4)	0.092
Atrial fibrillation	9 (11.1)	2 (14.3)	11 (11.6)	0.663
Postprocedure branch vessel dissection	6 (7.4)	1 (7.1)	7 (7.4)	1.0
Leg ischemia	2 (2.5)	—	2 (2.1)	1.0
DVT/PE	3 (3.7)	—	3 (3.2)	1.0
Wound infection	2 (2.5)	2 (14.3)	4 (4.2)	0.102
UTI	10 (12.3)	1 (7.1)	11 (11.6)	1.0
Respiratory infection	6 (7.4)	—	6 (6.3)	0.587
Sepsis	3 (3.7)	—	3 (3.2)	1.0
Prolonged intubation (>48 hours)	—	1 (7.1)	1 (1.0)	0.147
Reintubation	4 (4.4)	1 (7.1)	5 (5.3)	0.558
Bowel ischemia	4 (4.4)	1 (7.1)	5 (5.3)	0.558
Hematoma	11 (13.6)	2 (14.3)	13 (13.7)	1.0
Clostridium difficile infection	6 (7.4)	—	6 (6.3)	0.587
Brachial plexus injury	1 (1.2)	—	1 (1.0)	1.0
Reintervention	20 (24.7)	2 (14.3)	22 (23.2)	0.510
Endovascular procedures	16 (19.8)	2 (14.3)	18 (18.9)	
Open procedures	4 (4.9)	—	4 (4.2)	

Continuous data are presented as mean ± standard deviation or median (IQR); categorical data are given as the counts (percentage).

Abbreviations: Cr, creatinine; DVT/PE, deep venous thrombosis/pulmonary embolism; ICU, intensive care unit; IQR, interquartile range; MI, myocardial infarction; pRBC, packed red blood cell; TALE, thoracoabdominal aortic aneurysm life-altering events; TIA, transient ischemic attack; UTI, urinary tract infection.

a Developed SCI a mean of 4.2 ± 2.1 days after repair.

b Throughout entire hospital stay.

p<0.05 is considered significant.

**Figure 1.**
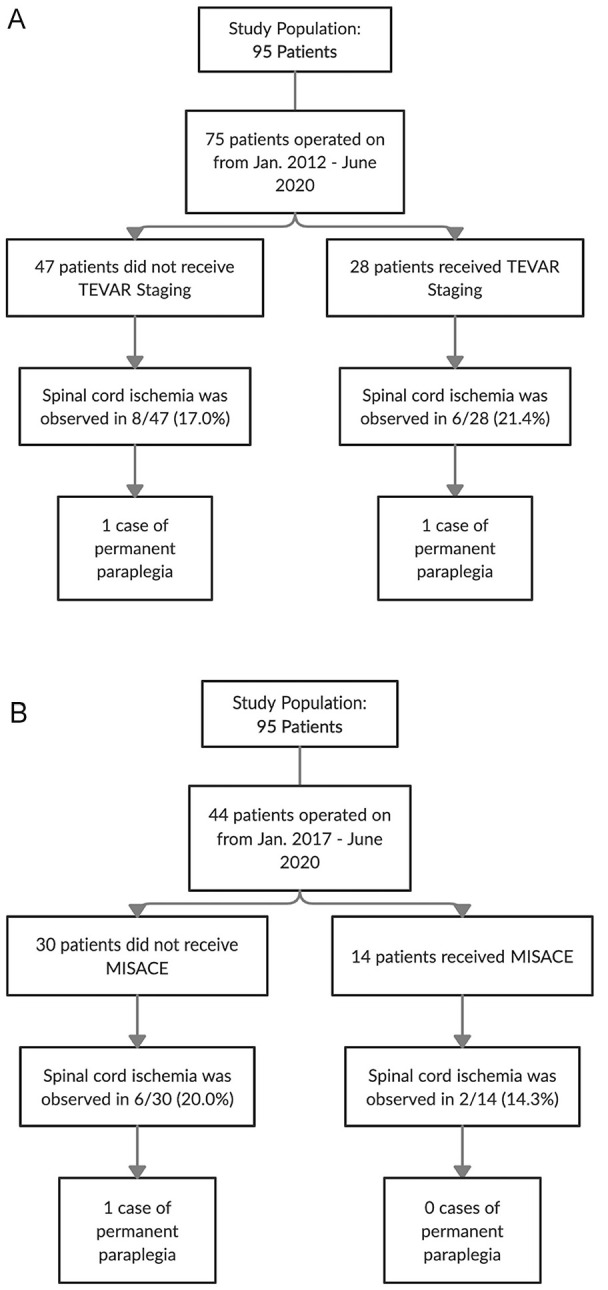
Flowchart of spinal cord ischemia and permanent paraplegia incidence in patients (A) with and without TEVAR staging and (B) with and without MISACE. TEVAR, thoracic endovascular aortic repair; MISACE, minimally invasive staged segmental artery coil embolization.

Eighteen (19%) patients developed SCI postoperatively with 4 (4.2%) permanent paraplegia (grade 3a-3c) cases (Table 3). Of the patients who received staged TEVAR, SCI developed in 21.4% (6/28; 1 permanent paraplegia and 1 permanent paraparesis) compared with 17.0% in those without staging (8/47; 1 permanent paraplegia and 3 permanent paraparesis, Figure 1A). SCI developed in 2 (14.3%) patients who received preoperative MISACE, with 1 case of permanent paraparesis and no permanent paraplegia. Spinal cord ischemia was observed in 6 (20.0%) non-MISACE patients, of whom 2 had permanent paraparesis and 1 had permanent paraplegia (Figure 1B). In-hospital mortality was observed in 4/18 patients (22.2%) who suffered SCI.

Renal dysfunction occurred in 6.3% of patients; 3.2% required temporary dialysis but no patients required permanent dialysis. Respiratory insufficiency necessitated 5 (5.3%) reintubations, whereas no patients required a tracheostomy. The incidence of the postoperative TALE was 15.8% (Table 3). Branch vessel dissection prior to discharge was observed in 10 arteries in 7 patients (7.4%) (n=1 each for left subclavian, CA, right hepatic, SMA, RR (Right renal), and left external iliac; n=2 for LR (Left Renal) and right external iliac each). Five of these required in-hospital reintervention with stenting.

In-hospital reintervention for the aorta and/or its branches was required in 22 patients (23.2%, 25 procedures: 21 percutaneous and 4 surgical). Surgical interventions included an axillary-axillary bypass to mitigate SCI (n=1) and 3 open artery repairs (left iliofemoral, right radial, and right external iliac artery repairs for iatrogenic injuries). Percutaneous procedures included creation of a type 1b endoleak to reverse SCI (n=3, successful in 1), restenting for branch instability 8.4% (stenosis, kink, occlusion, dissection, n=8). One patient required stenting of celiac, SMA, and LR arteries on his side after an incomplete emergent repair due to class III obesity that prevented imaging of those branches in the supine position. Two (2.1%) patients required branch coil embolization for bleeding (splenic artery and LR mid-pole artery) and 8 patients required endoleak repair (type 1b, n=4; type 3, n=4).

### Follow-up

**Figure 2.**
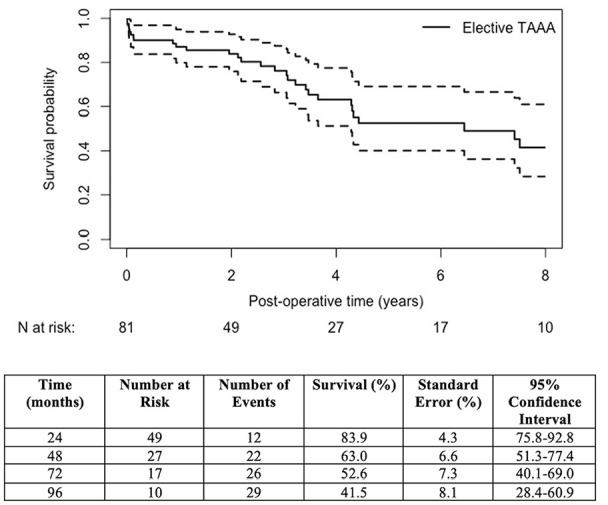
Kaplan-Meier estimates of elective patient survival after endovascular TAAA repair. TAAA, thoracoabdominal aortic aneurysm. ^a^Open splenohepatic bypass for a replaced hepatic artery leading to type III endoleak.

Clinical follow-up was 100% complete, with a mean follow-up of 3.6 ± 3.0 (range: 0-12) years during which 28 (32.6%) patients died. There was 1 confirmed aneurysm-related death: a rupture due to proximal aneurysm progression at the site of an unstented CA fenestration within the neck, 10 years after index repair in a patient that declined further intervention. Long-term survival for elective patients was 52.6% (95% confidence interval [CI], 40.1-69.0) at 5 years and 41.5% (95% CI, 28.4-60.9) at 8 years (Figure 2), whereas survival for the urgent/emergent cohort at 5 years was significantly lower, 33.7% (95% CI, 12.0-97.0; log-rank test, p<0.05). Freedom from all-cause mortality was 50.1% (95% CI, 38.4-65.4) at 5 years and 34.4% (95% CI, 22.5-52.8) at 8 years (Supplemental Figure 1). Table 4 highlights major complications in our series compared with open and EVAR literature.

**Table 4.** Comparison of Postoperative Outcomes Between Current Series and Contemporary Open/Endovascular TAAA Data.

Variable	Current series (n=95)	Ontario population data (endovascular, n=303)^4^	Systematic review (endovascular)^10^	Timaran et al (endovascular TAAA, n=396)^19,a^	Ontario population data (open, n=361)^4^	Systematic review (open)^10^	Coselli et al^3^ (open, n=3309)
Urgent/Emergent	14 (14.7%)	47 (15.5%)	—	0%	96 (26.6%)	—	723 (21.8%)
In-hospital mortality	9 (9.5%)	31 (10.2%)	7.4%	9 (2.3%)	64 (17.7%)	8.9%	237 (7.2%)
Spinal cord ischemia	18 (18.9%)	—	13.5%	—	—	7.4%	317 (9.6%)
Permanent paraplegia (grade 3a-3c)	4 (4.2%)	13 (4.3%)	5.2%	11 (2.8%)	13 (3.6%)	4.4%	97 (2.9%)
Stroke	5 (5.3%)	18 (5.9%)	2.7%	9 (2.3%)	18 (5.0%)	3.9%	98 (3.0%)
Permanent dialysis	0	8 (2.6%)	3.7%	7 (1.8%)	17 (4.7%)	3.8%	189 (5.7%)
TALE	15 (15.8%)	54 (17.8%)	—	—	93 (25.8%)	—	—
Acute kidney injury	6 (6.3%)	—	11.7%	29 (7.3%)	—	21.7%	406 (12.3%)
Transient dialysis	3 (3.2%)	21 (6.9%)	6.4%	—	45 (12.5%)	12.0%	61 (1.8%)
AMI	8 (8.4%)	18 (5.9%)	—	7 (1.8%)	22 (6.1%)	—	41 (1.2%)
Respiratory infection	6 (6.3%)	—	—	—	—	—	225 (6.8%)
Sepsis	3 (3.2%)	—	—	—	—	—	148 (4.5%)
Reintubation	5 (5.3%)	—	—	—	—	—	479 (14.5%)
Tracheostomy	0 (0)	—	—	—	—	—	281 (8.5%)

Continuous data are presented as mean ± standard deviation; categorical data are given as the counts (percentage).

Abbreviations: AMI, acute myocardial infarction; TAAA, thoracoabdominal aortic aneurysm; TALE, thoracoabdominal aortic aneurysm life-altering events.

a Data extracted from Supplemental Material and cohort did not include any urgent/emergent repairs.

During follow-up, 5 (5.8%) patients suffered a stroke on average 3.1 ± 2.2 years after repair and 3 (3.5%) patients required dialysis a mean of 1.3 ± 1.2 years after repair, none having required transient postoperative dialysis. Of the patients who required dialysis on follow-up, 2 had occluded an RR artery stent (1 had a solitary right kidney) and 1 following a subsequent open arch repair. Freedom from the composite TALE outcome was 47.1% (95% CI, 35.5-62.6) and 32.3% (95% CI, 20.8-50.1) at 5 and 8 years, respectively (Supplemental Figure 2).

Surveillance imaging was performed on 80/86 surviving patients (93%), with a mean time to last surveillance imaging postrepair of 2.6 ± 2.7 years. Stable aneurysm sac size was noted in 27 (33.8%) patients, decreased aneurysm sacs (<5 mm) in 36 (45%), and sac expansion (>5 mm) was noted in 16 (20.0%). In this latter group, 15 received reinterventions to resolve sac perfusion and 1 was treated conservatively. Postdischarge target vessel kink or dissection was observed in 6 (6.9%) patients with 4 requiring restenting. Long-term primary patency for successfully revascularized target vessels was 97.7% (333/341). On follow-up, 4 patients required reintervention for target vessel stenosis/thrombosis resulting in a secondary patency of 98.8%. Freedom from branch instability was 45.5% (95% CI, 33.8-61.3) at 5 years (Supplemental Figure 3).

**Figure 3.**
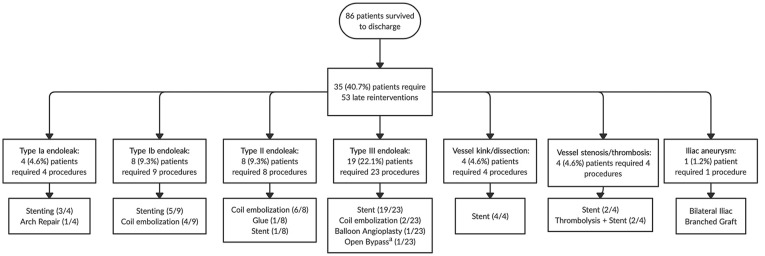
Flowchart of late reintervention indication and types of procedures executed, for a total of 53 (52 percutaneous and 1 open) reinterventions. ^a^Open splenohepatic bypass for a replaced hepatic artery leading to type III endoleak.

**Figure 4.**
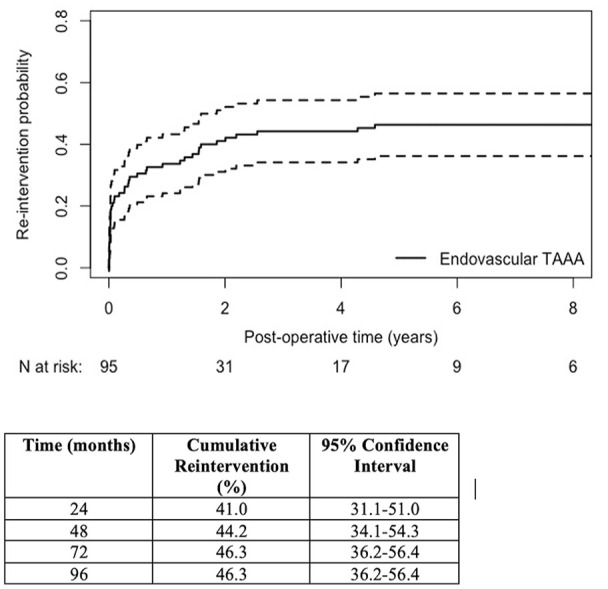
Cumulative probability of reintervention on the aorta and/or its branches after endovascular TAAA repair. TAAA, thoracoabdominal aortic aneurysm.

Forty-three patients had at least 1 endoleak identified on follow-up imaging, of which 31 (36.0%) received 44 procedures to resolve them. In total throughout follow-up, 35 (40.7%) patients required 53 late reinterventions (52 percutaneous and 1 open) for their aorta and/or visceral branches (Figure 3). Cumulative probability of reintervention was 46.3% (95% CI, 36.1-56.4) at both 5 and 8 years (Figure 4). Thirty day mortality after late reintervention was observed in 3 (8.5%) patients.

## Discussion

Endovascular TAAA repair remains complex and technically challenging with published single-center series follow-up ranging between 1.0 and 3.0 years.^10,20–22^ We present a 13 year experience with endovascular TAAA repair using F/BEVAR. Our cohort is a comorbid, older population who have all been assessed and turned down for open repair. Importantly, our data include the evolution of endovascular devices and stents, constant revision of procedural techniques, and patient selection, and include all urgent/emergent repair—displaying the adoption of evolving endovascular techniques by multiple surgeons for high-risk patients at a single center.

Endovascular TAAA repair has excellent technical success, with the celiac artery accounting for the largest number of unsuccessful target vessel revascularizations (7/13), also noted by others.^23^ As reported in literature, we too found target vessels to be occluded or stenosed between preoperative planning and the actual procedure,^24^ resulting in a decreased primary technical success rate. This did lead to a postoperative death due to rupture in a patient as the SMA was identified as occluded intraoperatively and could not be stented intraoperatively.

Elective in-hospital mortality was 7.4%, similar to reports from other high-volume endovascular centers and a pooled proportion value of 7% from a contemporary meta-analysis of endovascular TAAA repair (Table 4).^10,25,26^ Moreover, a 5% to 10% operative mortality rate has been shown in large open repair series, making F/BEVAR a competitive treatment option.^3,10^ An Ontario, Canada, population-based propensity-matched analysis comparing open versus endovascular TAAA repair reported a higher early mortality in the open cohort (17.4% vs 10.8%), further supporting the use of F/BEVAR.^4^ A 21.4% mortality rate was observed for urgent/emergent repairs; however, this rate is consistent among other reports of F/BEVAR for acute complex aortic disease.^27^

Long-term survival is superior in the elective cohort versus the urgent/emergent cohort, with overall 5 year survival numbers consistent with centers of excellence.^17,23,25^ Our mean follow-up is 3.6 years, with an 8 year overall survival of 34.4% with F/BEVAR, comparable to the 10 year survival of 36.8% observed after open repair from Coselli et al’s^3^ landmark study, despite the present series patients being on average 7 years older. At 8 years, there is a 7% higher survival rate in the elective cohort compared with the overall group, highlighting an important difference between cohorts operated electively and a consecutive cohort. In these older/comorbid patients, we demonstrate respectable long-term results, especially when compared with open repair literature.

Spinal cord ischemia remains a significant complication as endovascular series report a wide incidence between 4% and 33%.^10^ Our overall SCI rate was 18.9% with the vast majority in type I to III and type V repairs. Several reports have found extent of aneurysm, single-staged procedures, and occlusion of hypogastric arteries to be predictive of SCI.^28^ In the present series, 63.2% received extent II and III coverage (including appropriate seal zones), with 17/18 SCI cases being observed in this group. Increased aortic coverage as a risk factor for SCI was also reported by Kitpanit and colleagues.^29^ The rate of SCI in our urgent/emergent cohort was 28.6%, with a 40% SCI rate in those requiring extent II repair. These high rates can be attributed to symptomatic or ruptured aneurysms with hemodynamic instability and extensive aortic coverage performed in a single procedure. Nonetheless, only 4 patients suffered permanent paraplegia in the entire cohort. This rate is comparable to the 2.9% permanent paraplegia rate seen by Coselli et al^3^ after open TAAA repair and the 4.3% endovascular repair rate observed in the province of Ontario (Table 4).^4^

Minimization of SCI after endovascular TAAA repair is an ongoing area of research. Universally adopted techniques such as the use of prophylactic CSF drainage are now being questioned due to rates of serious complications^30^ and there is certainly a large amount of variance in techniques used to prevent SCI, even among high-volume centers.^31^ Nevertheless, a trend toward improvement is noted using a combination of preoperative MISACE, TEVAR staging, permissive hypertension, prophylactic CSF drainage, and intraoperative neuromonitoring.

O’Callaghan and others suggested that staging may reduce the incidence of SCI.^32,33^ Paradoxically, we had a higher rate of SCI (21.4%) in the staged compared with the nonstaged (17.0%) cohort. However, 24/28 (86%) of the staged patients received extent II coverage, which may have increased the risk of SCI and permanent paraplegia. Thoracic EVAR staging itself comes with a risk of interstage mortality.^23^ In our series, 2 patients presented with contained rupture and 1 with a symptomatic aneurysm requiring emergent repair with a T-branch graft between stages. The potential benefit of reduced SCI needs to be weighed against the risk of rupture for patients receiving staging. Data with larger sample sizes are needed to thoroughly evaluate this adjunctive technique in minimizing postoperative complications, compared with the use of MISACE.

Our practice further evolved in 2017 when the promising initial results of MISACE by Branzan et al^34^ showed no SCI after endovascular TAAA repair. We observed a trend in SCI improvement after MISACE with 2 cases of paraparetic SCI out of 14 MISACE-treated patients (14.3%), with no permanent paraplegia, while the rate of SCI in the non-MISACE cohort treated from 2017 onwards was 20.0%. Our current standard approach to reduce SCI in patients that require extensive coverage (<4 cm of above celiac axis) is a 3-stage approach of MISACE, TEVAR, followed by endovascular TAAA repair with spinal cord drainage, neuromonitoring, and permissive hypertension. Spinal cord ischemia rates require further improvements to reduce the postoperative SCI burden.

The low postoperative renal and pulmonary morbidity in our series are key advantages of endovascular TAAA repair. Despite the 38% incidence of preoperative stage III to IV chronic kidney disease (CKD), there were no cases of permanent dialysis and only 3 patients required transient dialysis, in keeping with rates of permanent dialysis of 0% to 8% reported by other centers.^10^ Conversely, high-volume open TAAA repair is associated with a 7.6% rate of postoperative dialysis, 5.7% of which is permanent.^3^ While renal dysfunction is a major morbidity of open repair,^35^ a low rate (3.5%) of dialysis in our long-term follow-up means F/BEVAR shows promise in operating on patients with preexisting CKD and reducing dialysis-dependent renal failure. Despite our significant incidence of COPD, no patients required a tracheostomy and only 5.3% required reintubation. In contrast, Coselli et al^3^ noted a 14.5% reintubation rate and a tracheostomy incidence of 8.5%. Our pulmonary-renal complication rates are similar to the ones seen at high-volume centers and confirm the analysis from multiple national database studies that suggest F/BEVAR leads to lower rates of perioperative morbidity, especially as it relates to renal and pulmonary complications.^17,36,37^

Postoperative rates of TALE, a composite outcome used to measure successful repair and recovery, were 15.8%, consistent with the 17.8% rate of postoperative TALE after endovascular repair in Ontario and are considerably lower than after open repair (25.8%) (Table 4).^4^ Long-term freedom from TALE was 47.1% at 5 years and 32.3% at 8 years and parallels freedom from mortality at 50.0% and 34.4%, respectively. Kang et al^38^ reported a lower “good outcome” (77%), their own measure of repair and recovery, compared with survival (88%) at 1 year in their endovascular TAAA cohort. Both terms are important because their components are viewed by patients as treatment failures; however, our longer follow-up data exhibit that major complications are rare in the follow-up period and postdischarge TALE is driven by mortality. Durability of TAAA repair by F/BEVAR was excellent with only 1 case of late aneurysm rupture identified at 10 years after primary repair, demonstrating that late mortality is infrequently due to aneurysm-related pathology.

Secondary procedures are common after endovascular TAAA repair, likely secondary to the multiple components required and our policy to aggressively treat endoleaks associated with aneurysm growth over 5 mm.^4,17,23^ Reinterventions were 98% percutaneous and 81% of them were to treat endoleaks, divided equally among fenestrations and branches. Despite the need for reintervention, long-term vessel patency is excellent at 98.8% overall at follow-up with only 4 patients requiring intervention for occlusion and/or stenosis. Moreover, most reinterventions occurred on the renal arteries for type III endoleaks, consistent with Mastracci’s report where the renal arteries were noted to be the most unstable with a high reintervention rate that did not affect long-term clinical outcomes.^39^ Rocha and colleagues^4^ reported a 14% 30 day mortality after reintervention for endovascular TAAA repair in Ontario, highlighting 1 of the limitations of endovascular repair over open repair; however, we observed this in only 8% of cases.

In the 20 years since the initial description of endovascular TAAA repair, similar results are achieved and even improved results compared with the 70 year evolution of open repair. A summary of TAAA outcomes from population-based studies, systematic reviews, and the largest open series is compared in Table 4. While patient cohorts differ with respect to the incidence of connective tissue disorders and comorbidities, it is clear that endovascular TAAA repair has overcome its early technical challenges and outcomes are likely to continue to improve with better bridging stents and strategies to reduce SCI.

Limitations of this study include the retrospective nature, technical evolution of the procedure, and the “learning curve” associated with a complex procedure with several surgeons. While the mean follow-up was only 3.0 years due to inclusion of recent repairs (June 2020), we demonstrate some of the longest follow-up to date, a maximum of 12 years and Kaplan-Meier estimates to 8 years. However, further long-term data with close imaging follow-up are needed to better characterize durability and need for late reintervention. The biggest limitation is the modest and diverse sample size.

## Conclusion

This series demonstrates a single-center evolution of endovascular TAAA repair. Mortality and SCI, while higher compared with referral centers, are on par with systematic reviews and compare favorably to population-based open/EVAR TAAA repair data from Ontario. Importantly, we demonstrate the renal and pulmonary advantages of endovascular TAAA repair. While further refinements in techniques continue, long-term survival and branch patency was excellent, with very few branch occlusions. We demonstrate safety in high-risk patients, even as the procedure and technology evolved. Further improvements in short-term and long-term outcomes of endovascular TAAA repair rival open repair and should be subject to prospective comparative studies.

## Supplemental Material

sj-tiff-1-jet-10.1177_15266028241229005 – Supplemental material for Thoracoabdominal Aortic Aneurysm Repair Using Fenestrated and Branched Endovascular Grafts for High-Risk Patients: Evolving yet Safe Supplemental material, sj-tiff-1-jet-10.1177_15266028241229005 for Thoracoabdominal Aortic Aneurysm Repair Using Fenestrated and Branched Endovascular Grafts for High-Risk Patients: Evolving yet Safe by Daniyal N. Mahmood, Rodolfo Rocha, Maral Ouzounian, Kong Teng Tan, Samantha M. Forbes, Jennifer C-Y. Chung and Thomas F. Lindsay in Journal of Endovascular Therapy

sj-tiff-2-jet-10.1177_15266028241229005 – Supplemental material for Thoracoabdominal Aortic Aneurysm Repair Using Fenestrated and Branched Endovascular Grafts for High-Risk Patients: Evolving yet Safe Supplemental material, sj-tiff-2-jet-10.1177_15266028241229005 for Thoracoabdominal Aortic Aneurysm Repair Using Fenestrated and Branched Endovascular Grafts for High-Risk Patients: Evolving yet Safe by Daniyal N. Mahmood, Rodolfo Rocha, Maral Ouzounian, Kong Teng Tan, Samantha M. Forbes, Jennifer C-Y. Chung and Thomas F. Lindsay in Journal of Endovascular Therapy

sj-tiff-3-jet-10.1177_15266028241229005 – Supplemental material for Thoracoabdominal Aortic Aneurysm Repair Using Fenestrated and Branched Endovascular Grafts for High-Risk Patients: Evolving yet Safe Supplemental material, sj-tiff-3-jet-10.1177_15266028241229005 for Thoracoabdominal Aortic Aneurysm Repair Using Fenestrated and Branched Endovascular Grafts for High-Risk Patients: Evolving yet Safe by Daniyal N. Mahmood, Rodolfo Rocha, Maral Ouzounian, Kong Teng Tan, Samantha M. Forbes, Jennifer C-Y. Chung and Thomas F. Lindsay in Journal of Endovascular Therapy

## References

[1] Chuter TA Gordon RL Reilly LM , et al. An endovascular system for thoracoabdominal aortic aneurysm repair. J Endovasc Ther. 2001;8(1):25–33. 11220464 10.1177/152660280100800104

[2] Ouzounian M LeMaire SA Weldon S , et al. Open repair of thoracoabdominal aortic aneurysm: step-by-step. Oper Tech Thorac Cardiovasc Surg. 2018;23(1):2–20. doi:10.1053/j.optechstcvs.2018.07.002.

[3] Coselli JS LeMaire SA Preventza O , et al. Outcomes of 3309 thoracoabdominal aortic aneurysm repairs. J Thorac Cardiovasc Surg. 2016;151(5):1323–1337. doi:10.1016/j.jtcvs.2015.12.050. 26898979

[4] Rocha RV Lindsay TF Austin PC , et al. Outcomes after endovascular versus open thoracoabdominal aortic aneurysm repair: a population-based study. J Thorac Cardiovasc Surg. 2021;161(2):516–527. doi:10.1016/j.jtcvs.2019.09.148. 31780062

[5] Parodi JC Palmaz JC Barone HD. Transfemoral intraluminal graft implantation for abdominal aortic aneurysms. Ann Vasc Surg. 1991;5(6):491–499. doi:10.1007/BF02015271. 1837729

[6] Giannopoulos S Kokkinidis DG Armstrong EJ. Long term outcomes of endovascular vs open surgical repair for abdominal aortic aneurysms: a meta-analysis of randomized trials. Cardiovasc Revasc Med. 2020;21(10):1253–1259. doi:10.1016/j.carrev.2020.02.015. 32265128

[7] Powell JT Sweeting MJ Ulug P , et al. Meta-analysis of individual-patient data from EVAR-1, DREAM, OVER and ACE trials comparing outcomes of endovascular or open repair for abdominal aortic aneurysm over 5 years. Br J Surg. 2017;104(3):166–178. doi:10.1016/j.jvs.2017.03.403. 28160528 PMC5299468

[8] Li B Khan S Salata K , et al. A systematic review and meta-analysis of the long-term outcomes of endovascular versus open repair of abdominal aortic aneurysm. J Vasc Surg. 2019;70(3):954–969.e30. doi:10.1016/j.jvs.2019.01.076. 31147117

[9] Greenberg RK Lu Q Roselli EE , et al. Contemporary analysis of descending thoracic and thoracoabdominal aneurysm repair: a comparison of endovascular and open techniques. Circulation. 2008;118(8):808–817. doi:10.1161/CIRCULATIONAHA.108.769695. 18678769

[10] Rocha RV Lindsay TF Friedrich JO , et al. Systematic review of contemporary outcomes of endovascular and open thoracoabdominal aortic aneurysm repair. J Vasc Surg. 2020;71(4):1396–1412. doi:10.1016/j.jvs.2019.06.216. 31690525

[11] Ferrer C Cao P De Rango P , et al. A propensity-matched comparison for endovascular and open repair of thoracoabdominal aortic aneurysms. J Vasc Surg. 2016;63(5):1201–1207. doi:10.1016/j.jvs.2015.10.099. 26776896

[12] Rocha RV Friedrich JO Elbatarny M , et al. A systematic review and meta-analysis of early outcomes after endovascular versus open repair of thoracoabdominal aortic aneurysms. J Vasc Surg. 2018;68(6):1936–1945. doi:10.1016/j.jvs.2018.08.147. 30470373

[13] Oderich GS Forbes TL Chaer R , et al. Reporting standards for endovascular aortic repair of aneurysms involving the renal-mesenteric arteries. J Vasc Surg. 2021;73(suppl 1):4S–52S. doi:10.1016/j.jvs.2020.06.011. 32615285

[14] Chaikof EL Dalman RL Eskandari MK , et al. The Society for Vascular Surgery practice guidelines on the care of patients with an abdominal aortic aneurysm. J Vasc Surg. 2018;67(1):2–77.e2. doi:10.1016/j.jvs.2017.10.044. 29268916

[15] Safi HJ Miller CC. Spinal cord protection in descending thoracic and thoracoabdominal aortic repair. Ann Thorac Surg. 1999;67(6):1937–1939; discussion 1953–1958. doi:10.1016/s0003-4975(99)00397-5. 10391343

[16] Addas JAK Mafeld S Mahmood DN , et al. Minimally Invasive Segmental Artery Coil Embolization (MISACE) prior to endovascular thoracoabdominal aortic aneurysm repair. Cardiovasc Intervent Radiol. 2022;45(10):1462–1469. doi:10.1007/s00270-022-03230-y. 35927497

[17] Oderich GS Ribeiro M Reis de Souza L , et al. Endovascular repair of thoracoabdominal aortic aneurysms using fenestrated and branched endografts. J Thorac Cardiovasc Surg. 2017;153(2):S32–S41. doi:10.1016/j.jtcvs.2016.10.008. 27866781

[18] Geisbüsch S Stefanovic A Koruth JS , et al. Endovascular coil embolization of segmental arteries prevents paraplegia after subsequent thoracoabdominal aneurysm repair: an experimental model. J Vasc Surg. 2014;147(1):220–226. doi:10.1016/j.jtcvs.2013.09.022. PMC3918675 24220154

[19] Timaran CH Oderich GS Tenorio ER , et al. Expanded use of preloaded branched and fenestrated endografts for endovascular repair of complex aortic aneurysms. Eur J Vasc Endovasc Surg. 2021;61(2):219–226. doi:10.1016/j.ejvs.2020.11.001 33262091 10.1016/j.ejvs.2020.11.001

[20] Anderson JL Adam DJ Berce M , et al. Repair of thoracoabdominal aortic aneurysms with fenestrated and branched endovascular stent grafts. J Vasc Surg. 2005;42(4):600–607. doi:10.1016/j.jvs.2005.05.063. 16242539

[21] Verhoeven EL Tielliu IF Bos WT , et al. Present and future of branched stent grafts in thoraco-abdominal aortic aneurysm repair: a single-centre experience. Eur J Vasc Endovasc Surg. 2009;38(2):155–161. doi:10.1016/j.ejvs.2009.05.002. 19523863

[22] Walker J Kaushik S Hoffman M , et al. Long-term durability of multibranched endovascular repair of thoracoabdominal and pararenal aortic aneurysms. J Vasc Surg. 2019;69(2):341–347. doi:10.1016/j.jvs.2018.04.074. 30683193

[23] Eagleton MJ Follansbee M Wolski K , et al. Fenestrated and branched endovascular aneurysm repair outcomes for type II and III thoracoabdominal aortic aneurysms. J Vasc Surg. 2016;63(4):930–942. doi:10.1016/j.jvs.2015.10.095. 26792544

[24] Gallitto E Faggioli G Spath P , et al. The risk of aneurysm rupture and target visceral vessel occlusion during the lead period of custom-made fenestrated/branched endograft. J Vasc Surg. 2020;72(1):16–24. doi:10.1016/j.jvs.2019.08.273. 32063442

[25] Verhoeven EL Katsargyris A Bekkema F , et al. Editor’s choice—ten-year experience with endovascular repair of thoracoabdominal aortic aneurysms: results from 166 consecutive patients. Eur J Vasc Endovasc Surg. 2015;49(5):524–531. doi:10.1016/j.ejvs.2014.11.018. 25599593

[26] Guillou M Bianchini A Sobocinski J , et al. Endovascular treatment of thoracoabdominal aortic aneurysms. J Vasc Surg. 2012;56(1):65–73. doi:10.1016/j.jvs.2012.01.008. 22560310

[27] Juszczak MT Vezzosi M Khan M , et al. Endovascular repair of acute juxtarenal and thoracoabdominal aortic aneurysms with surgeon-modified fenestrated endografts. J Vasc Surg. 2020;72(2):435–444. doi:10.1016/j.jvs.2019.10.056. 31882311

[28] Eagleton MJ Shah S Petkosevek D , et al. Hypogastric and subclavian artery patency affects onset and recovery of spinal cord ischemia associated with aortic endografting. J Vasc Surg. 2014;59(1):89–94. doi:10.1016/j.jvs.2013.07.007. 24188715

[29] Kitpanit N Ellozy SH Connolly PH , et al. Risk factors for spinal cord injury and complications of cerebrospinal fluid drainage in patients undergoing fenestrated and branched endovascular aneurysm repair. J Vasc Surg. 2021;73(2):399–409. doi:10.1016/j.jvs.2020.05.070. 32640318

[30] Plotkin A Han SM Weaver FA , et al. Complications associated with lumbar drain placement for endovascular aortic repair. J Vasc Surg. 2021;73(1):1513–1524. doi:10.1016/j.jvs.2020.08.150. 33053415

[31] Aucoin VJ Eagleton MJ Farber MA , et al. Spinal cord protection practices used during endovascular repair of complex aortic aneurysms by the U.S. Aortic Research Consortium. J Vasc Surg. 2021;73(1):323–330. doi:10.1016/j.jvs.2020.07.107. 32882346

[32] O’Callaghan A Mastracci TM Eagleton MJ. Staged endovascular repair of thoracoabdominal aortic aneurysms limits incidence and severity of spinal cord ischemia. J Vasc Surg. 2015;61(2):347–354. doi:10.1016/j.jvs.2014.09.011. 25449006

[33] Bertoglio L Katsarou M Loschi D , et al. Elective multistaged endovascular repair of thoraco-abdominal aneurysms with fenestrated and branched endografts to mitigate spinal cord ischaemia. Eur J Vasc Endovasc Surg. 2020;59(4):565–576. doi:10.1016/j.ejvs.2019.10.003. 31870689

[34] Branzan D Etz CD Moche M , et al. Ischaemic preconditioning of the spinal cord to prevent spinal cord ischaemia during endovascular repair of thoracoabdominal aortic aneurysm: first clinical experience. EuroIntervention. 2018;14(7):828–835. doi:10.4244/EIJ-D-18-00200. 29969429

[35] Cajas -Monson L D’Oria M Tenorio E , et al. Effect of renal function on patient survival after endovascular thoracoabdominal and pararenal aortic aneurysm repair. J Vasc Surg. 2021:74(1):13–19. doi:10.1016/j.jvs.2020.11.040. 33340697

[36] Tsilimparis N Perez S Dayama A , et al. Endovascular repair with fenestrated-branched stent grafts improves 30-day outcomes for complex aortic aneurysms compared with open repair. Ann Vasc Surg. 2013;27(3):267–273. doi:10.1016/j.avsg.2012.05.022. 23403330

[37] Rosenfeld ES Macsata RA Lala S , et al. Open surgical repair of juxtarenal abdominal aortic aneurysms in the elderly is not associated with increased thirty-day mortality compared with fenestrated endovascular grafting. J Vasc Surg. 2021;73(4):1139–1147. doi:10.1016/j.jvs.2020.08.121. 32919026

[38] Kang PC Bartek MA Shalhub S , et al. Survival and patient-centered outcome in a disease-based observational cohort study of patients with thoracoabdominal aortic aneurysm. J Vasc Surg. 2019;70(5):1427–1435. doi:10.1016/j.jvs.2019.02.033. 31147133 PMC7064148

[39] Mastracci TM Greenberg RK Eagleton MJ , et al. Durability of branches in branched and fenestrated endografts. J Vasc Surg. 2013;57(4):926–933; discussion 933. doi:10.1016/j.jvs.2012.09.071. 23433817

