# Supplementary figures and images for: Thoracoabdominal Aortic Aneurysm Repair Using Fenestrated and Branched Endovascular Grafts for High-Risk Patients: Evolving yet Safe
Source: J Endovasc Ther. 2024 Feb 10;32(6):1994–2006. doi: 10.1177/15266028241229005 (PMC12598073; doi:10.1177/15266028241229005)

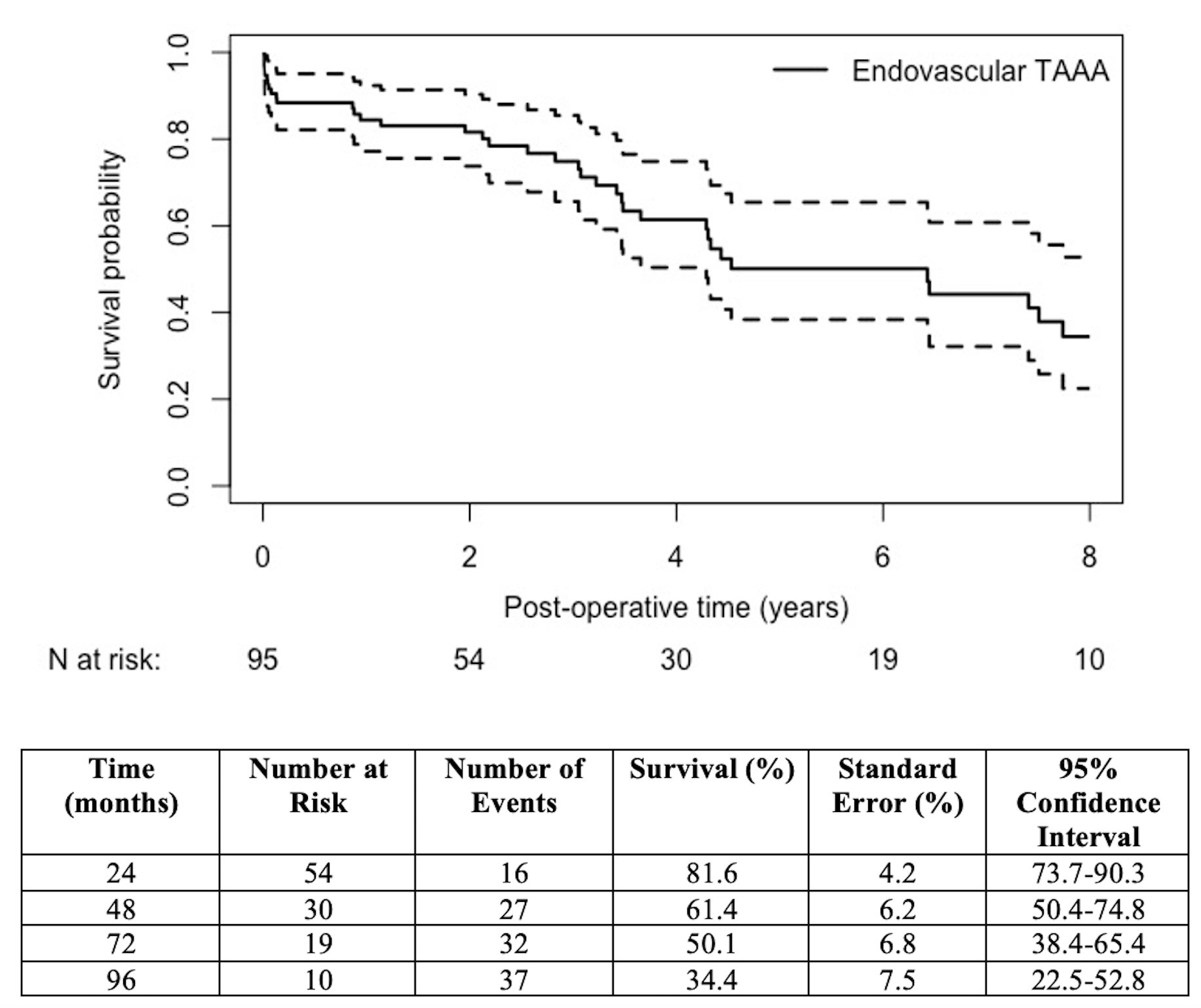

Supplement: sj-tiff-1-jet-10.1177_15266028241229005 – Supplemental material for Thoracoabdominal Aortic Aneurysm Repair Using Fenestrated and Branched Endovascular Grafts for High-Risk Patients: Evolving yet Safe [file sj-tiff-1-jet-10.1177_15266028241229005.tiff]

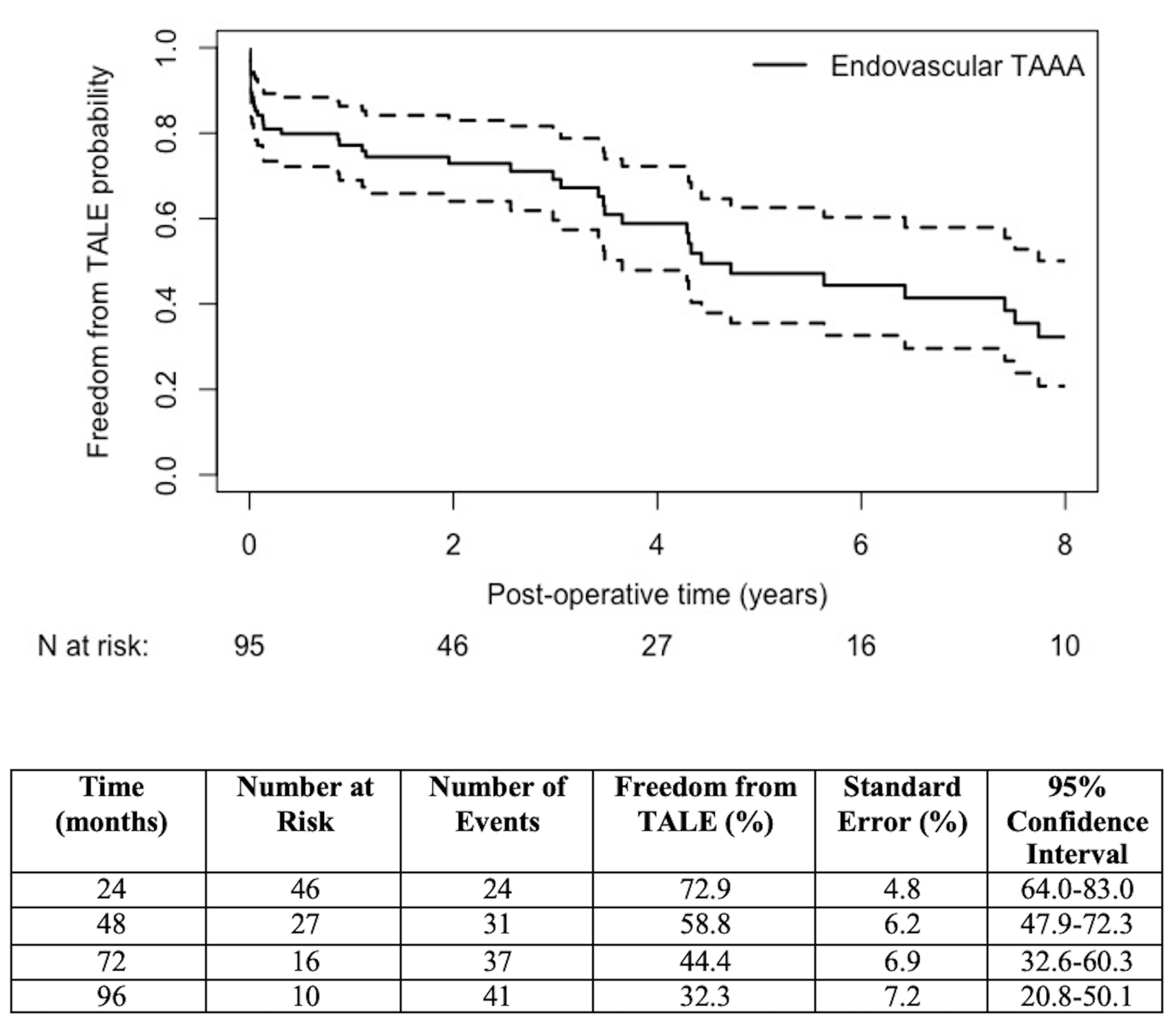

Supplement: sj-tiff-2-jet-10.1177_15266028241229005 – Supplemental material for Thoracoabdominal Aortic Aneurysm Repair Using Fenestrated and Branched Endovascular Grafts for High-Risk Patients: Evolving yet Safe [file sj-tiff-2-jet-10.1177_15266028241229005.tiff]

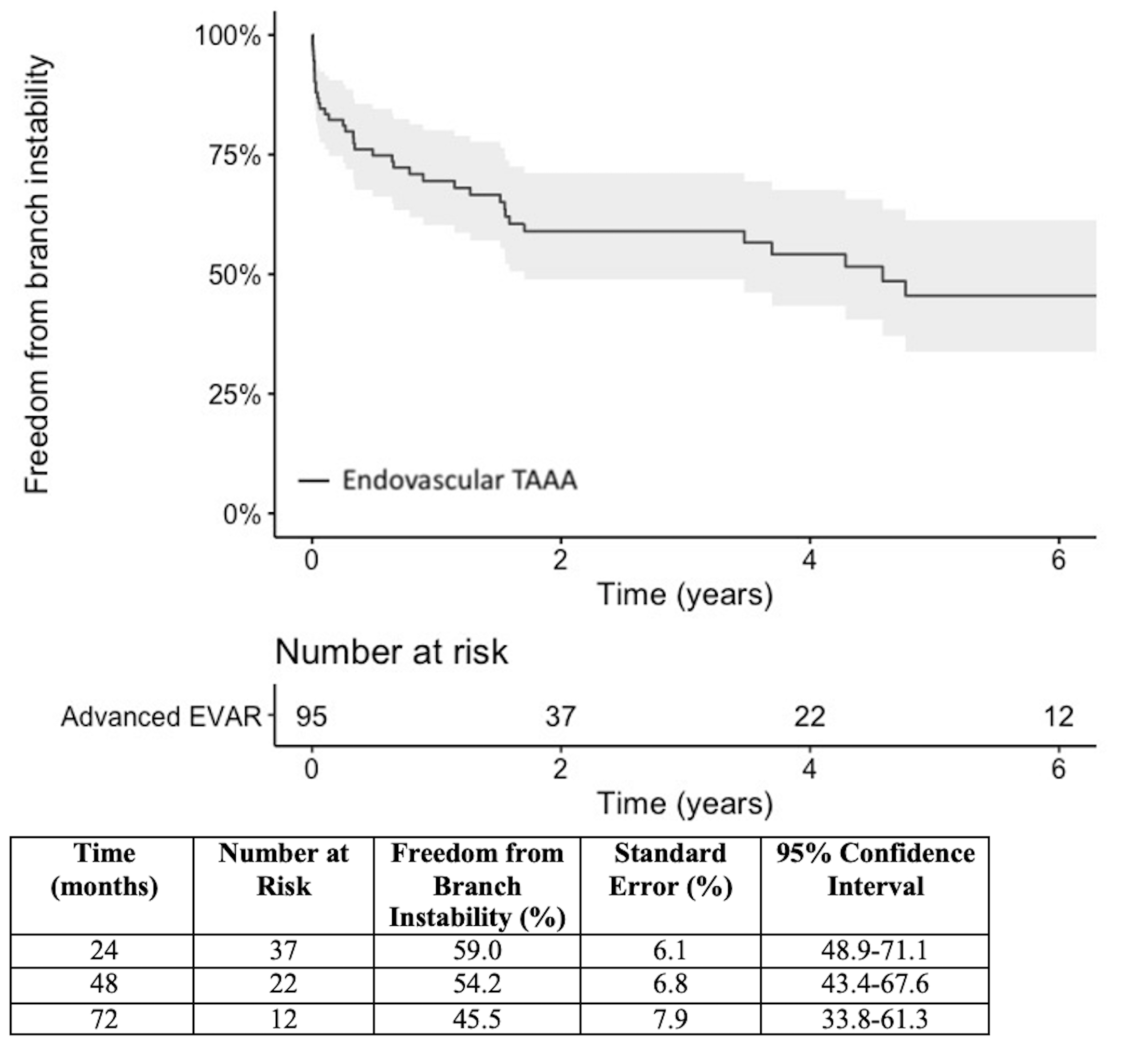

Supplement: sj-tiff-3-jet-10.1177_15266028241229005 – Supplemental material for Thoracoabdominal Aortic Aneurysm Repair Using Fenestrated and Branched Endovascular Grafts for High-Risk Patients: Evolving yet Safe [file sj-tiff-3-jet-10.1177_15266028241229005.tiff]
